# Multiplex Real-Time PCR Assay Targeting Eight Parasites Customized to the Korean Population: Potential Use for Detection in Diarrheal Stool Samples from Gastroenteritis Patients

**DOI:** 10.1371/journal.pone.0166957

**Published:** 2016-11-18

**Authors:** Eun Jeong Won, Soo Hyun Kim, Seung Jung Kee, Jong Hee Shin, Soon Pal Suh, Jong Yil Chai, Dong Wook Ryang, Myung Geun Shin

**Affiliations:** 1 Departments of Laboratory Medicine, Chonnam National University Medical School and Chonnam National University Hospital, Gwangju, Republic of Korea; 2 Department of Parasitology and Tropical Medicine, Seoul National University College of Medicine, Seoul, Republic of Korea; 3 Brain Korea 21 Plus Project, Chonnam National University Medical School, Gwangju, Republic of Korea; 4 Environmental Health Center for Childhood Leukemia and Cancer, Chonnam National University Medical School and Chonnam National University Hwasun Hospital, Hwasun, Jeollanam-do, Republic of Korea; Hebrew University, ISRAEL

## Abstract

Intestinal parasitic diseases occur worldwide and can cause diarrhea or gastroenteritis; however, their diagnosis is quite difficult, especially in low-endemism countries. We developed a multiplex real-time PCR assay for detection of eight intestinal parasites and prospectively evaluated it for patients with gastroenteritis. The assay targeted *Cryptosporidium parvum*, *Giardia lamblia*, *Entamoeba histolytica*, *Blastocystis hominis*, *Dientamoeba fragilis*, *Clonorchis sinensis*, *Metagonimus yokogawai*, and *Gymnophalloides seoi*. Performance characteristics were evaluated based on recovery after DNA extraction, analytical sensitivity, specificity, reproducibility, cross-reactivity, and interference characteristics. Clinical performance was validated against microscopy on 123 diarrheal samples. The assay demonstrated strong correlations between DNA concentrations and C_t_ values (R^2^, 0.9924–0.9998), and had a high PCR efficiency (83.3%–109.5%). Polymerase chain reactions detected as few as 10–30 copies of genomic DNA, and coefficient of variance was 0–7%. There was no cross-reactivity to the other 54 microorganisms tested. Interference occurred only in presence of high concentrations of erythrocytes or leukocytes. This assay had a higher correct identification rate (100.0% vs. 90.2%) and lower incorrect ID rate (0.0% vs. 9.8%) when compared to microscopy. Overall, this assay showed a higher sensitivity (100.0%; 95% confidence interval [CI] of 80.5–100.0) than microscopy (29.4%; 95% CI 10.31–55.96), and the specificity levels were comparable for both methods (100.0%; 95% CI 96.58–100.0). This newly developed multiplex real-time PCR assay offers a potential use for detecting intestinal parasitic pathogens customized to the Korean population.

## Introduction

Intestinal parasitic diseases have been detected among the populations of all countries, and the World Health Organization estimates that 3.5 billion people worldwide are infected with some type of an intestinal parasite [1]. In humans, infection with an intestinal parasite can be asymptomatic, but some cases present with diarrhea and other signs of gastroenteritis. In contrast to bacterial or viral pathogens, it is not easy to diagnose the parasitic pathogen responsible for a diarrhea case. Microscopic examination of stool samples for the detection of cysts, oocysts, and trophozoites remains the diagnostic method of choice for many laboratories [2]; however, the method requires technical expertise, and it is laborious; it can also be insensitive at low levels of infection. Meanwhile, molecular approaches involving polymerase chain reaction (PCR) are becoming increasingly available for detecting intestinal parasites, and these molecular methods demonstrate excellent sensitivity and specificity with respect to conventional methods such as microscopy [3].

The most common etiological causes of intestinal infections are species of *Cryptosporidium*, *Blastocystis* and *Entamoeba*, as well as *Giardia lamblia* and *Dientamoeba fragilis* in a worldwide basis [4]. However, the frequency of parasitic disease could be variable according to the region studied. In Korea, it is known that *Clonorchis sinensis* and *Metagonimus yokogawai* are the most common intestinal parasites [5, 6], and *Gymnophalloides seoi* from infected oysters is also frequently found in southern coastal areas and causes gastroenteritis after ingestion [7, 8]. Thus, this study aimed i) to develop a multiplex qPCR assay for eight major intestinal parasites known to cause gastroenteritis in the Korean population, and ii) to validate this assay against conventional microscopy using diarrheal stool samples obtained from patients with gastroenteritis in Korea.

## Materials and Methods

### Primer design

The major parasites causing gastroenteritis selected for this study were: Five protozoa including *Cryptosporidium parvum*, *Giardia lamblia*, *Entamoeba histolytica*, *Blastocystis hominis*, *Dientamoeba fragilis*, and three trematodes including *Clonorchis sinensis*, *Metagonimus yokogawai*, and *Gymnophalloides seoi*. The target genes of each parasite were available from GenBank and were selected as follows (S1 Table): The 18S rRNA genes of *B*. *hominis* (GenBank: EU482085.1), *E*. *histolytica* (GenBank: X65163.1), and *D*. *fragilis* (GenBank: JQ677163.1); Cryptosporidium oocyst wall protein gene of *C*. *parvum* (GenBank: AB089292.1); beta-giardin gene of *G*. *lamblia* (GenBank: XM_001705373.1); and cytochrome c oxidase subunit 1 regions of *G*. *seoi* (GenBank: AF096234.3), *M*. *yokogawai* (GenBank: AB470519.1), and *C*. *sinensis* (GenBank: FJ381664.2), respectively. The sets of primers were designed using Primer 3 software (http://frodo.wi.mit.edu/primer3/). Each primer for amplification was selected with size ranging from 18 to 27 base pairs (optimally 20 base pairs), melting temperature ranging from 50°C to 58°C, and GC content ranging from 36% to 50%.

### Amplification, TA cloning, and clone screening for positive control

PCR amplifications were performed using conventional real-time thermo cycler platform, CFX96 (Bio-Rad, Hercules, CA, USA) and a 20 μL reaction volume containing 10 μL of parasite reaction mixture (1 unit of Hot start Taq polymerase, each 2 mM of dNTPs, 40 mM of Tris-Cl, 100 mM KCl, 0.02% Tween 20, 6 mM of MgCl_2_), each 1 μL of primers (forward and reverse, 10 pmol/μL), and 3 μL of genomic DNA of parasite (1 ng/μL), and 5 μL of deionized water. The PCR reaction was established by gradient annealing using *G*. *seoi* and *C*. *sinensis* as follows: pre-denaturing at 95°C for 10 min, 35 cycles of denaturation at 95°C with 30 sec, gradient annealing from 70°C to 50°C for 30 sec, and extension at 72°C with 30–60 sec and final extension at 72°C for 10 min. Prior to subsequent TA cloning or sequencing procedure, PCR product purification was performed according to the manufacturer’s manual (GeneAll Biotechnology, Cat.#103–150, Seoul, Korea), and was run on 1.5% agarose gel (0.5X TBE buffer, 250 V, 30 min). Purified PCR products were cloned via a commercially available T&A Cloning Vector Kit (Cat.#RC013, Real Biotech Corporation, Banqiao City, Taiwan). The positive control plasmids containing 380 bp PCR products for *G*. *seoi*, 511 bp PCR products for *B*. *homins*, 533 bp PCR products for *C*. *parvum*, 464 bp PCR products for *G*. *lamblia*, 540 bp PCR products for *E*. *histolytica*, 360 bp PCR products for *M*. *yokogawai* and *C*. *sinensis* were constructed by a commercially available T&A Cloning Vector Kit (Cat.#RC013, Real Biotech). Positive transformants carrying the vector were ampicillin resistant, and the clones with the inserted DNA fragment grew into white colonies on solid media containing X-gal (with IPTG) and ampicillin. Colony PCR was performed on the white colonies to screen for clones containing the inserted target gene after extracting DNA using a plasmid mini prep kit (GeneAll Biotechnology), and according to the manufacturer’s protocol. To check whether the target genes were inserted in colony plasmids, we performed the PCR again with the same primers and PCR reaction mixtures as aforementioned. As a representative example, in S1 Fig, the PCR products from *E*. *histolytica* target sequences obtained from 12 cloned colonies and their sequencing results are shown.

### Determining the optimized probes for multiplex qPCR

To optimize the probes for multiplex qPCR, PCR was performed using 10 μL of parasite reaction mixture, primer and probe mixture including each 500 nM of primers (forward and reverse) and 250 nM of probe, and 5 μL of positive controls for parasite (100 fg). PCR conditions were as follows: 50°C for 2 min, 95°C for 10 min, and 45 cycles of denaturation at 95°C with 15 sec, annealing at 60°C for 1 min, extension at 72°C with 30 sec. The proper combination of primers and probes was determined by the cycle threshold (Ct) values.

### PCR efficiency test

PCR reactions were performed 6 times using 10-fold dilutions of genomic DNA from 100 fg to 10 ag. The value of slope was determined by equation of the correlation between the DNA contents and their Ct values. PCR efficiency (%) was calculated by the below equations or using the efficiency calculator at the Life Technologies website (Carlsbad, CA, USA) (https://www.lifetechnologies.com/kr/ko/home/brands/thermo-scientific/molecular-biology/molecular-biology-learning-center/molecular-biology-resource-library/thermo-scientific-web-tools/qpcr-efficiency-calculator.html.)

PCR efficiency (E) = 10−1/slope

PCR efficiency (%) = (E−1) × 100%

### Analytical sensitivity and precision test

Total DNA from sample containing 3 × 10^8^ clones/mL approximately were diluted in 10-fold increments from 100 pg to 10 ag for evaluating analytic sensitivity as the limit of detection (LoD). Each 100 ag of parasite DNA was considered as LoD with a minimum of 30 copies. For data analysis, the melting curve and Ct values were selected as evaluation parameters. Tests were repeated 24 times to validate precision by calculation of coefficients of variation (CV). The value of LoD was determined as the concentration of positive samples that produced a positive signal 95% of the time according to the EP17-A, CLSI guidelines [9].

### Cross-reactivity and interference test

Genomic DNA of 54 pathogens including 27 bacteria/candida, 11 parasites, and 16 viruses were screened for cross-reactivity. Interference effect was also evaluated according to various concentrations of the major compounds, such as erythrocytes (0–20%), leukocytes (0–20%), bilirubin (0–2 μg/mL), bile salts (0–1 mg/mL), heparin (0–0.2 IU), acetaminophen (0–200 μg/mL), and ibuprofen (0–200 μg/mL), respectively. After DNA extraction, the PCR procedure was performed as above. Every test was repeated 4-times for each parasite.

### Validation of the multiplex qPCR assay with conventional microscopy and using gastroenteritis stool samples

A total of 123 fresh, unpreserved stool samples were tested. The study cohort consisted of (i) diarrheal stool samples obtained from patients with gastroenteritis at Chonnam National University Hospital (n = 117), and (ii) positive samples (n = 6) prepared by mixing stool samples from healthy controls (for the purpose of laboratory quality control) with ova or cysts of the parasites *G*. *lamblia* (n = 2), *E*. *histolytica* (n = 2), or *G*. *seoi* (n = 2). Samples were analyzed within 12 h by conventional microscopic method using formalin-ether concentration with additional special stains, if necessary (iodine stain, and modified acid fast stain). Briefly, the staining involved having 1 g fresh or formalinized feces suspended in 10 mL of 10% formalin in ParaTubes (KS Corporation, Sungnam, Korea), followed by centrifugation at 500 g for 1 min. After centrifugation, the inner tube with filtered debris was discarded. Then, 3 mL ethyl acetate was added to the cone-bottomed tube. The tube was centrifuged at 500 g for 10 min, the supernatant decanted, and the top plug of the debris rimmed with an applicator stick. The remaining sediment was diluted in a few drops of 10% formalin and 20 μL was placed onto a slide for parasite examination. For multiplex qPCR assay, total DNA from 200 mg of each stool sample was extracted with a DNA stool minikit (Qiagen, Hilden, Germany) and eluted in a final volume of 200 μL according to the manufacturer’s recommendations. PCR amplifications were performed using a CFX96 RT-PCR system (Bio-Rad) and a 20-μL reaction volume containing 10 μL of parasite reaction mixture, 5 μL of primer/probe mixture (each 500 nM of forward and reverse primers, 250 nM of probe) and 5 μL of DNA solution. The PCR proceeded as follows: pre-denaturing at 95°C for 10 min, 45 cycles of denaturation at 95°C with 15 sec, annealing and elongation at 60°C with 1 min [10]. Final identification was made to i) concordant results between two results obtained from multiplex PCR and conventional microscopy, and ii) sequencing result using individual primers when discordant results were obtained between the two methods. Determination of correct or incorrect identification results were based on this final identification.

### Statistical analysis

Chi-square or Fisher exact test was performed to compare the performance of the multiplex qPCR with the conventional method. Pearson correlation coefficients were used to examine the correlation between the DNA contents and their Ct values. Student t-test was used for comparison of laboratory characteristics of gastroenteritis patients enrolled in this study. A *P* <0.05 indicated significance for all analyses. All statistical analyses were performed by using PASW version 18.0 (SPSS Inc., Chicago, IL, USA).

### Ethics Statement

Collection of the fecal samples for this study was conducted in accordance with the guidelines and approval of the Institutional Review Board (IRB) of Chonnam National University Hospital (IRB CNUH-2015-052). Given the nature of the project, a waiver of consent was granted for the remaining samples, and no information was used that could lead to patient identification.

## Results

### Development of a multiplex qPCR assay for the major parasites causing gastroenteritis

All DNA samples for the eight parasites were obtained with clone screening. The DNA sequences were confirmed by sequencing and subsequent NCBI database 100% identity match with organism sequences from GenBank (S1 Table and S1 Fig). Optimized sequences of probes were determined by having Ct values of less than 30; the optimized combination of primers and probes are described in Table 1.

**Table 1:** Established combination of primers and probes using the multiplex real-time PCR assay for the major parasites causing gastroenteritis.

Target organism	Forward primer sequence (5'-3')	Reverse primer sequence (5'-3')	Optimized probe sequence (5'-3')	Target	Accession number of target sequence
*Gymnophalloides seoi*	AGTGTTGTTTGGGCTCATCA	AACCTTAATCGGTGGGAACA	FAM-TGTTTATGGTTGGTTGATGTTAAGACTTCGGT-black hole quencher 1	CO1	AF096234.3
*Blastocystis hominis*	GGAGAGGGAGCCTGAGAGAT	AACATTGTTCCGCATTGTGA	VIC-ATCCTGACACAGGGAGGTAGTG-non fluorescent quencher MGB	18s rRNA	EU482085.1
*Cryptosporidium parvum*	TGTGTTCAATATCTCCCTGCAAA	GCATGTCGATTCAATTTGTCA	Quasar670-CCTCCTGGATTCAATTTGTCA-black hole quencher plus	Cowp 1	AB089292.1
*Giardia lamblia*	GAGGTCAAGAAGTCCGCCG	CAAGGGACTTGCGGAAGTTT	FAM-ACGATCAAGGAGGAGATCGA-black hole quencher plus	beta-giardin	XM_001705373.1
*Entamoeba histolytica*	GCGGACGGCTCATTATAACA	TGTCGTGGCATCCTAACTCA	VIC-AAATGGCCAATTCATTCAATG-non fluorescent quencher MGB	18s rRNA	X65163.1
*Metagonimus yokogawai*	CTATGGCGGTTTAGTGTTGGC	ACTGCCGTCTTCAAATCCAG	Quasar670-TGGGCGCATCACATGTTTATGGTG-black hole quencher plus	CO1	AB470519.1
*Clonorchis sinensis*	GGTGGTTTGAGCTCATCATATGT	CGAGTTCCAGCAAGCATATATAATC	FAM-CGGTTACTATGATTATAGGTGTGCC-black hole quencher 1	CO1	FJ381664.2
*Dientamoeba fragilis*	TTAGAACCTTAGACAACGGATGTCTTG	TGTGCATTCAAAGATCGAACTTATC	VIC-TGTGATAAGCGGCTAGAATTGC-non fluorescent quencher MGB	18s rRNA	JQ677163.1

CO1 = cytochrome c oxidase subunit 1; Cowp = Cryptosporidium oocyst wall protein

All of targets showed excellent correlation for DNA concentration and Ct values (R^2^, 0.9924–0.9998) along with high PCR efficiencies ranging from 83.3% to 109.5% (Table 2 and S2 Fig). The PCR method allowed detection of as few as 10 copies (30 ag) for *G*. *lamblia*, *M*. *yokogawai*, and about 30 copies (100 ag) for *C*. *parvum*, *E*. *histolytica*, *D*. *fragilis*, *B*. *hominis*, *C*. *sinensis* and *G*. *seoi* (Table 3). The CV values for the replicate experiments were below 10% (0%– 7%) for all eight parasites. No amplification was observed for DNA extracted from 54 other microorganisms (S2 Table). Interference analysis revealed that the DNA samples were identifiable under all test conditions, except those with a high concentration (20%) of erythrocytes or leukocytes and 0.2 IU of heparin (S3 Table).

**Table 2:** PCR efficiency (%) obtained from slope of dependence of DNA concentrations on threshold cycle (Ct) values.

Target organism	Average C_t_ values as a function of DNA concentrations					R^2^	Slope	Efficiency (%)
	100 fg	10 fg	1 fg	100 ag	10 ag			
***Cryptosporidium parvum***	26.0	29.5	33.1	37.3	41.1	0.999	-3.8	83.3
***Giardia lamblia***	27.4	30.8	34.4	37.3	41.1	0.998	-3.39	97.1
***Entamoeba histolytica***	26.5	30.0	33.4	36.7	39.9	1	-3.34	99.1
***Dientamoeba fragilis***	27.9	31.5	35.2	38.2	41.7	0.999	-3.43	95.6
***Blastocystis hominis***	30.3	33.7	37.2	40.9	43.0	0.992	-3.25	103.2
***Clonorchis sinensis***	25.7	29.3	32.8	36.1	39.5	1	-3.43	95.6
***Metagonimus yokogawai***	25.8	29.4	32.7	36.4	39.6	0.999	-3.47	94.3
***Gymnophalloides seoi***	30.9	34.1	37.6	40.8	43.2	0.996	-3.12	109.5

**Table 3:** Analytical sensitivity (limit of detection, LoD) and analytical precision of the multiplex qPCR assay.

		Average C_t_ values depending on the copy number of target DNA						
Target organism	Performances	30,000 copies	3,000 copies	300 copies	30 copies	10 copies	5 copies	N.C.
***Cryptosporidium parvum***								
	Average C_t_ value	26.05	29.2	32.9	36.26	39.31	39.41	-
	SD	0.11	0.22	0.45	0.43	2.22	1.58	-
	CV	0.41%	0.74%	1.37%	1.17%	5.65%	4.00%	-
	Detection rate	100%	100%	100%	100%	67%	38%	-
***Giardia lamblia***								
	Average C_t_ value	27.46	30.77	34.15	37.24	40.98	39.26	-
	SD	0.23	0.27	0.46	0.75	2.23	0.97	-
	CV	0.83%	0.86%	1.34%	2.01%	5.45%	2.47%	-
	Detection rate	100%	100%	100%	100%	100%	33%	0%
***Entamoeba histolytica***								
	Average C_t_ value	26.52	30.02	33.36	36.94	39.28	39.01	-
	SD	0.17	0.22	0.39	1.69	1.72	1.77	-
	CV	0.64%	0.74%	1.16%	4.58%	4.37%	4.55%	-
	Detection rate	100%	100%	100%	96%	54%	25%	0%
***Dientamoeba fragilis***								
	Average C_t_ value	27.73	31.15	34.7	37.53	40.72	41.44	-
	SD	0.39	0.32	0.65	0.73	2.11	1.95	-
	CV	1.40%	1.04%	1.86%	1.96%	5.19%	4.71%	-
	Detection rate	100%	100%	100%	96%	63%	71%	0%
***Blastocystis hominis***								
	Average C_t_ value	30.47	34.08	37.29	40.63	42.25	40.77	-
	SD	0.41	0.51	0.59	0.82	1.55	1.4	-
	CV	1.35%	1.51%	1.58%	2.01%	3.68%	3.43%	-
	Detection rate	100%	100%	100%	100%	46%	17%	0%
***Clonorchis sinensis***								
	Average C_t_ value	25.73	29.28	32.23	36.23	39.47	39.2	-
	SD	0.12	0.28	0.35	1.07	2.68	1.84	-
	CV	0.48%	0.97%	1.09%	2.96%	6.78%	4.70%	-
	Detection rate	100%	100%	100%	100%	79%	50%	0%
***Metagonimus yokogawai***								
	Average C_t_ value	25.88	29.39	32.24	35.65	38.38	39.51	-
	SD	0.09	0.17	0.65	1.77	1.31	2.11	-
	CV	0.35%	0.57%	2.02%	4.96%	3.42%	5.34%	-
	Detection rate	100%	100%	100%	100%	100%	58%	0%
***Gymnophalloides seoi***								
	Average C_t_ value	30.15	33.94	37.2	40.14	41.64	40.21	-
	SD	0.45	0.33	0.42	0.76	1.56	1.66	-
	CV	1.51%	0.97%	1.12%	1.89%	3.74%	4.12%	-
	Detection rate	100%	100%	100%	100%	54%	13%	0%

Ct = threshold cycle; CV = coefficient of variation; N.C. = negative control; SD = standard deviation.

### Validation of performance of the multiplex qPCR assay on the major parasites causing gastroenteritis

**Fig 1:**
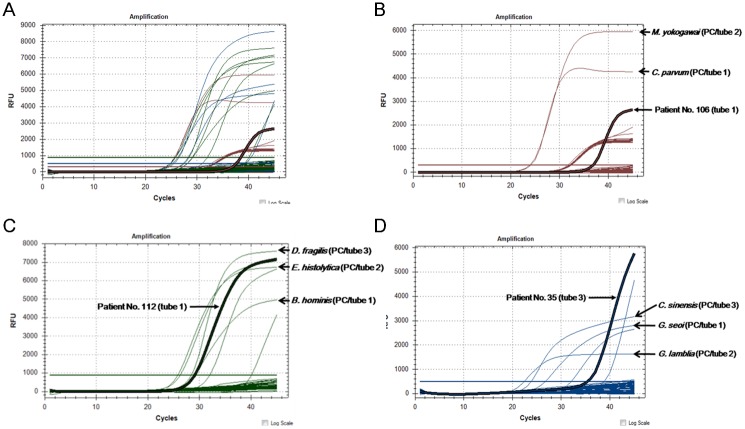
Representative reaction curves of the multiplex real-time PCR assay. A) Multiple reaction curves indicate amplification of the positive controls and clinical samples obtained from patients with gastroenteritis. B) Positive curves of two positive controls for *Cryptosporidium parvum* (tube 1) and *Metagonimus yokogawai* (tube 2) were observed in the Quasar 670 channel. Patient No. 106’s sample showed a positive curve within tube 1 according to Quasar 670 signals. C) Positive curves of two positive controls for *Blastocystis hominis* (tube 1), *Entamoeba histolytica* (tube 2), and *Dientamoeba fragilis* (tube 3) were observed in the VIC/HEX channel. Patient No. 112’s sample showed a positive curve within tube 1 according to the VIC/HEX signals. D) Positive curves of two positive controls for *Gymnophalloides seoi* (tube 1), *Giardia lamblia* (tube 2), and *Clonorchis sinensis* (tube 3) were observed in the FAM channel. Patient No. 35’s sample showed a positive curve within tube 3 according to FAM signals.

Of 117 clinical diarrheal samples, *B*. *hominis* (n = 8, 6.8%) was the most frequently detected, followed by *C*. *sinensis* (n = 2, 1.7%), and *C*. *parvum* (n = 1, 0.9%). Among the 123 samples, which included six positive controls, the multiplex qPCR assay detected 17 samples containing parasites: 8 clinical samples of *B*. *hominis*, one of *C*. *parvum*, 3 of *C*. *sinensis*, and all 6 of the positive controls (*G*. *lamblia*, *E*. *histolytica*, and *G*. *seoi*) (Table 4 and Fig 1). The microscopic examinations detected 5 samples: one of *C*. *sinensis*, 2 of *G*. *lamblia*, and 2 of *E*. *histolytica*. Two positive samples of *G*. *seoi*, however, were not found by microscopy. The multiplex qPCR assay showed a higher correct identification rate than did the microscopic examination (100.0% vs. 90.2%, respectively; *P* = 0.003; Table 4). Moreover, incorrect results were obtained more frequently with microscopic examination than with the multiplex qPCR assay (9.8% vs. 0.0%, respectively). The multiplex qPCR assay exhibited a sensitivity of 100.0%; this statistic had a 95% confidence interval (CI) of 80.5–100.0. In addition, the multiplex qPCR assay exhibited a specificity of 100.0% (95% CI 96.58–100.0), a positive predictive value of 100.0% (95% CI 81.49–100.0), and a negative predictive value of 100.0% (95% CI 96.58–100.0). Microscopic examination exhibited a 29.4% sensitivity (95% CI 10.31–55.96), a 100.0% specificity (95% CI 96.58–100.0), a 100.0% positive predictive value (95% CI 47.82–100.0), and a negative predictive value of 89.8% (95% CI 82.91–94.63; Table 5).

**Table 4:** Comparison of microscopic examination and multiplex qPCR in terms of detection of the major parasites causing gastroenteritis, in stool samples from patients with diarrhea.

Final identification	No. of specimens	Microscopy		Multiple qPCR	
		Correct ID	Incorrect ID	Correct ID	Incorrect ID
***Blastocystis hominis***	8	0	8	8	0
***Cryptosporidium parvum***	1	0	1	1	0
***Clonorchis sinensis***	2	1	1	2	0
***Entamoeba histolytica*** ^a^	2	2	0	2	0
***Giardia lamblia*** ^a^	2	2	0	2	0
***Gymnophalloides seoi*** ^a^	2	0	2	2	0
**Negative for parasites**	106	106	0	106	0
**Total No. (%)**	123	111 (90.2)	12 (9.8)	123 (100.0)	0 (0.0)

^a^ The six positive controls include 2 of *Entamoeba histolytica*, 2 of *Giardia lamblia*, and 2 of *Gymnophalloides seoi*.

Abbreviations: ID, identification.

**Table 5:** Diagnostic performance from microscopic examination and multiplex qPCR in terms of detection of the major parasites causing gastroenteritis in stool samples from patients with diarrhea.

Methods	Results	No. of specimens with			Diagnostic performance [95% confidence interval]			
		Positive for the parasites	Negative for the parasites	Subtotal	SEN	SPE	PPV	NPV
Microscopy	Positive	5	0	5				
	Negative	12	106	118				
	Subtotal	17	106	123	29.40%	100.00%	100.00%	89.80%
					[10.31–55.96]	[96.58–100.0]	[47.82–100.0]	[82.91–94.63]
Multiplex qPCR	Positive	17	0	17				
	Negative	0	106	106				
	Subtotal	17	106	123	100.00%	100.00%	100.00%	100.00%
					[80.5–100.0]	[96.58–100.0]	[81.49–100.0]	[96.58–100.0]

Abbreviations: NPV, negative predictive value; PPV, positive predictive value; SEN, sensitivity; SPE, specificity

## Discussion

Over the last decade, application of multiplex qPCR to detection of intestinal parasitic infections has compared favorably against microscopy in a variety of settings [11–13]. The present study developed a new multiplex qPCR assay for detection of parasites causing gastroenteritis, targeting the eight major pathogens that frequently occur in Korea: *C*. *parvum*, *G*. *lamblia*, *E*. *histolytica*, *B*. *hominis*, *D*. *fragilis*, *C*. *sinensis*, *M*. *yokogawai*, and *G*. *seoi*. This assay exhibited excellent linearity, high analytical sensitivity, and high analytical specificity. Moreover, clinical performance of the assay was validated on stool samples from patients with gastroenteritis in Korea.

The multiplex qPCR assay described in this study provides an alternative detection system for the most clinically important diarrhea-causing protozoa that cannot easily be found in stool microscopic examinations without special staining [14, 15]. Reports also indicate that parasite-specific DNA assays are more sensitive than microscopy or ELISA for detecting major intestinal protozoa such as *C*. *parvum*, *G*. *lamblia*, and *E*. *histolytica* [16–18]. Although both microscopy and qPCR assay could detect all positive controls for *G*. *lamblia* and *E*. *histolytica*, a specimen containing *C*. *parvum* was identified only by qPCR, but not by microscopy. In serial follow-up samples obtained from this AIDS patient over the subsequent three days, the qPCR assay could detect *C*. *parvum* in all three samples. However, microscopic examination with trichrome staining could find *C*. *parvum* only in the Day 3 sample. Like microscopy, a cryptosporidium antigen test by an enzyme-linked immunosorbent assay (ELISA; Ridascreen) could not detect *C*. *parvum* in the first submitted stool sample (data not shown). This makes the multiplex qPCR assay a sensitive detection method for globally significant protozoan pathogens.

It is notable that *B*. *hominis* was the most common agent observed in diarrhea specimens obtained from the gastroenteritis patients. Blastocystosis is generally associated with nonspecific symptoms such as diarrhea and abdominal pain [19]. Some researchers have suggested that *Blastocystis* species are associated with the irritable bowel syndrome [20, 21]. However, the pathophysiology of *Blastocystis* infections in Korea remains undefined, and highly sensitive quantitative tools would increase our understanding of *Blastocystis* infections. Elghareeb et al. found a wide range in diagnostic sensitivity between different *Blastocystis* detection methods [22]. Xenic *in vitro* cultures (XIVC) of *Blastocystis* exhibited a heightened positivity of 22.8%, compared to 12.3% with trichrome staining, 10.0% with formalin-ether concentration, 6.0% with an iodine-stained smear, and 3.5% with a direct smear [22]. Several PCR assays targeting the SSU rRNA gene have been previously developed for detection and discrimination among *Blastocystis* isolates directly in stool samples, and these assays have been more sensitive than XIVC [23]. The present study also supported this, finding that 6.8% of diarrheal samples were positive for *Blastocystis* according to the multiplex qPCR assay, whereas conventional microscopy with trichrome staining could not detect any such samples. We encourage further epidemiological and pathophysiological study of *Blastocystis* infections in Korea.

Several trematodes frequently found in the Korean population were included in the targets of this multiplex qPCR assay. Two samples with *C*. *sinensis* were identified using the multiplex qPCR, whereas one sample was missed using microscopy, irrespective of retesting. The diagnosis of this infection has mainly depended on detection of eggs in feces; however, this approach has poor sensitivity in cases of mild infection. It is also difficult to differentiate *C*. *sinensis* eggs from eggs of other parasitic trematodes with similar morphological characteristics, including *Opisthorchis* spp. and *M*. *yokogawai*. *C*. *sinensis* is carcinogenic in humans, and it was reclassified as a Group 1 biological carcinogen in 2009 [24]. Any evidence of clonorchiasis may lead to recommendation of chemotherapy in endemic areas. These results suggest that a precise diagnosis by multiplex qPCR may also be beneficial for patients infected with *C*. *sinensis*, which is the most common human liver fluke in Korea [25]. Moreover, this multiplex qPCR assay had a potential to detect trematodes other than *C*. *sinensis*, which present similar morphological characteristics of the eggs. Some researchers have developed PCR assays targeting *C*. *sinensis* and the other fish-borne trematodes such as *Opisthorchis viverrini* [26]. Instead of *O*. *viverrini* scarcely found in Korea, we focused on *M*. *yokogawai*, which is the most important heterophyid fluke with respect to general public health in Korea [27]. Although there were no cases of *M*. *yokogawai* in the submitted diarrheal specimens, we could expect the potential use of this assay considering that large and small rivers in the eastern and southern coastal areas of Korea were endemic foci of *M*. *yokogawai* infection [27, 28]. It is noteworthy that this is the first multiplex qPCR assay, as far as we know, to detect intestinal trematodes prevalent in Korea such as *M*. *yokogawai and G*. *seoi* using diarrheal specimens. *G*. *seoi* was first discovered in a Korean woman with acute pancreatitis and gastrointestinal discomfort in 1988 [7, 29]. In the southern to western coastal areas of Korea, the egg-positive rates of *G*. *seoi* are 9.3–24.1%, and the number of recovered parasite specimens is 37,489 in total (2,205 per person) [8]. Among the positive controls, two samples with *G*. *seoi* were identified with the multiplex qPCR assay, but not by microscopy. These presented Ct values of over 39 (39.79 and 39.14) in the qPCR assay and this could result in false negative by microscopy. According to the multiplex qPCR assay, the infection with *G*. *seoi* affects less than 1% of Korean patients with diarrhea. However, considering the high prevalence of *G*. *seoi* in Korea, the relation between *G*. *seoi* and various abdominal diseases needs to be further studied.

The advantage of this multiplex qPCR assay is sensitivity to *Cryptosporidium*, *Blastocystis*, and *Dientamoeba* without reliance on another diagnostic approach (such as modified acid-fast or trichrome staining). The LoDs for the parasites in this assay were found to range from 30–100 ag/μL. Because no eggs or larvae were detected by microscopy, we cannot say with certainty whether our failure to detect infections by microscopy was caused by low infection levels. A reasonable and biologically plausible alternative explanation is that qPCR can detect DNA at any lifecycle stage, whereas typical microscopy is optimized for detection of a single stage. For these reasons, qPCR may replace a large variety of species-specific microscopic methods. This assay also included an internal control to assess for possible inhibition of qPCR, and thereby identifying false negative samples.

This study had certain limitations. This study was to validate a newly developed multiplex qPCR assay targeting eight intestinal parasites using diarrheal samples obtained from gastroenteritis patients. Because of the small number of parasitic infections in the submitted specimens, the clinical validation might be not sufficient for *M*. *yokogwai* and *D*. *fragilis*. The current study could not find any evidence of *D*. *fragilis* infection in patients with diarrhea, although *D*. *fragilis* has been recently highlighted with possibility of pathogens present in irritable bowel syndrome [30]. The molecular diagnosis of this parasite has been recommended, as direct smear preparations are difficult due to the lack of a cyst stage and the permanent staining method is laborious and time-consuming [30]. Further research should be performed on a Korean population regarding the relation between *D*. *fragilis* infection and various abdominal diseases. Also, clinical performance to detect *M*. *yokogawai* should be further validated with a large number of patients including *M*. *yokogawai*-infected individuals and a long-duration study considering that prevalence of *M*. *yokogawai* infection could differ by season and geography. Furthermore, evaluation in a wide range of study cohorts including those with irritable bowel syndrome or inflammatory bowel diseases could be helpful for the application of this diagnostic in various clinical environments.

Multiplex qPCR may offer better sensitivity to intestinal parasitic infections than microscopic observations can offer. Laboratories using only microscopy to detect parasites may be missing up to 60% of positive cases, considering that the microscopy assays reported here detected only seven of 20 test-positive samples. This method represents a sensitive and specific assay for detecting gastrointestinal parasitism, with broad implications for community-based therapies and methods for assessing efficacy of treatment.

## Supporting Information

S1 Fig Representative figure for the cloning procedure to obtain positive controls of *E*. *histolytica*. PCR products (517 bp size) obtained from 12 cloned colonies (A) and their sequencing results showing 100% match with sequences of *E*. *histolytica* (GenBank: X65163.1).
(TIF)

S2 Fig Plots of serial dilutions of genomic DNA for the eight parasites. PCRs were performed on six 10-fold dilutions from 100 fg to 10 ag. Good correlations between the DNA concentration and the threshold cycle (Ct) values are seen.
(TIF)

S1 Table Details of amplification primers of the parasites for making positive controls. (PDF)

S2 Table Cross-reactivity test of the multiplex real-time PCR assay in relation to various microorganisms including 27 bacteria/candida, 11 parasites, and 16 viruses. (PDF)

S3 Table Interference analysis of different concentrations of interferents to positive controls. (PDF)

S4 Table Sensitivity and specificity of microscopic examination and multiplex qPCR in detection of the targeted parasites in this study. (PDF)

## References

[1] World Health Organization. The global burden of disease: 2004 update. Available: http://www.who.int/healthinfo/global_burden_disease/2004_report_update/en/

[2] McHardy IH, Wu M, Shimizu-Cohen R, Couturier MR, Humphries RM. Detection of intestinal protozoa in the clinical laboratory. J Clin Microbiol. 2014; 52:712–20. 10.1128/JCM.02877-13 24197877 PMC3957779

[3] Verweij JJ. Application of PCR-based methods for diagnosis of intestinal parasitic infections in the clinical laboratory. Parasitology. 2014; 141:1863–72. 10.1017/S0031182014000419 24780241

[4] Fletcher SM, Stark D, Harkness J, Ellis J. Enteric protozoa in the developed world: a public health perspective. Clin Microbiol Rev. 2012; 25:420–9. 10.1128/CMR.05038-11 22763633 PMC3416492

[5] Chai JY, Lee SH. Food-borne intestinal trematode infections in the Republic of Korea. Parasitol Int. 2002; 51:129–54. 12113752 10.1016/s1383-5769(02)00008-9

[6] Kim TS, Cho SH, Huh S, Kong Y, Sohn WM, Hwang SS, et al A nationwide survey on the prevalence of intestinal parasitic infections in the Republic of Korea, 2004. Korean J Parasitol. 2009; 47:37–47. 10.3347/kjp.2009.47.1.37 19290090 PMC2655332

[7] Chai JY, Choi MH, Yu JR, Lee SH. *Gymnophalloides seoi*: a new human intestinal trematode. Trends Parasitol. 2003; 19:109–12. 12643990 10.1016/s1471-4922(02)00068-5

[8] Guk SM, Park JH, Shin EH, Kim JL, Lin A, Chai JY. Prevalence of *Gymnophalloides seoi* infection in coastal villages of Haenam-gun and Yeongam-gun, Republic of Korea. Korean J Parasitol. 2006; 44:1–5. 10.3347/kjp.2006.44.1.1 16514291 PMC2532639

[9] CLSI. Protocols for determination of limits of detection and limits of quantitation; approved guideline CLSI document EP17-A, Wayne (PA): CLSI; 2012.

[10] Won EJ, Kim J, Ryang DW. Evaluation of modified formalin-ether concentration method using para tube in clinical settings. Ann Lab Med. 2015; 35:445–8. 10.3343/alm.2015.35.4.445 26131417 PMC4446584

[11] ten Hove R, Schuurman T, Kooistra M, Moller L, van Lieshout L, Verweij JJ. Detection of diarrhoea-causing protozoa in general practice patients in the Netherlands by multiplex real-time PCR. Clin Microbiol Infect. 2007; 13:1001–7. 10.1111/j.1469-0691.2007.01788.x 17714523

[12] Bruijnesteijn van Coppenraet LE, Wallinga JA, Ruijs GJ, Bruins MJ, Verweij JJ. Parasitological diagnosis combining an internally controlled real-time PCR assay for the detection of four protozoa in stool samples with a testing algorithm for microscopy. Clin Microbiol Infect. 2009; 15:869–74. 10.1111/j.1469-0691.2009.02894.x 19624500

[13] Nazeer JT, El Sayed Khalifa K, von Thien H, El-Sibaei MM, Abdel-Hamid MY, Tawfik RA, et al Use of multiplex real-time PCR for detection of common diarrhea causing protozoan parasites in Egypt. Parasitol Res. 2013; 112:595–601. 10.1007/s00436-012-3171-8 23114927

[14] de Wit MA, Koopmans MP, Kortbeek LM, van Leeuwen NJ, Vinje J, van Duynhoven YT. Etiology of gastroenteritis in sentinel general practices in the netherlands. Clin Infect Dis. 2001; 33:280–8. 10.1086/321875 11438890

[15] Stark D, Barratt JL, van Hal S, Marriott D, Harkness J, Ellis JT. Clinical significance of enteric protozoa in the immunosuppressed human population. Clin Microbiol Rev. 2009; 22:634–50. 10.1128/CMR.00017-09 19822892 PMC2772358

[16] Verweij JJ, Schinkel J, Laeijendecker D, van Rooyen MA, van Lieshout L, Polderman AM. Real-time PCR for the detection of *Giardia lamblia*. Mol Cell Probes. 2003; 17:223–5. 14580396 10.1016/s0890-8508(03)00057-4

[17] Morgan UM, Pallant L, Dwyer BW, Forbes DA, Rich G, Thompson RC. Comparison of PCR and microscopy for detection of *Cryptosporidium parvum* in human fecal specimens: clinical trial. J Clin Microbiol. 1998; 36:995–8. 9542924 10.1128/jcm.36.4.995-998.1998 PMC104676

[18] Mirelman D, Nuchamowitz Y, Stolarsky T. Comparison of use of enzyme-linked immunosorbent assay-based kits and PCR amplification of rRNA genes for simultaneous detection of *Entamoeba histolytica* and *E*. *dispar*. J Clin Microbiol. 1997; 35:2405–7. 9276425 10.1128/jcm.35.9.2405-2407.1997 PMC229977

[19] Cirioni O, Giacometti A, Drenaggi D, Ancarani F, Scalise G. Prevalence and clinical relevance of *Blastocystis hominis* in diverse patient cohorts. Eur J Epidemiol. 1999; 15:389–93. 10414382 10.1023/a:1007551218671

[20] Surangsrirat S, Thamrongwittawatpong L, Piyaniran W, Naaglor T, Khoprasert C, Taamasri P, et al Assessment of the association between Blastocystis infection and irritable bowel syndrome. J Med Assoc Thai. 2010; 93:S119–24. 21280524

[21] Jimenez-Gonzalez DE, Martinez-Flores WA, Reyes-Gordillo J, Ramirez-Miranda ME, Arroyo-Escalante S, Romero-Valdovinos M, et al Blastocystis infection is associated with irritable bowel syndrome in a Mexican patient population. Parasitol Res. 2012; 110:1269–75. 10.1007/s00436-011-2626-7 21870243

[22] Elghareeb AS, Younis MS, El Fakahany AF, Nagaty IM, Nagib MM. Laboratory diagnosis of *Blastocystis* spp. in diarrheic patients. Trop Parasitol. 2015; 5:36–41. 10.4103/2229-5070.149919 25709951 PMC4326992

[23] Stensvold CR, Arendrup MC, Jespersgaard C, Molbak K, Nielsen HV. Detecting Blastocystis using parasitologic and DNA-based methods: a comparative study. Diagn Microbiol Infect Dis. 2007; 59:303–7. 10.1016/j.diagmicrobio.2007.06.003 17913433

[24] de Martel C, Ferlay J, Franceschi S, Vignat J, Bray F, Forman D, et al Global burden of cancers attributable to infections in 2008: a review and synthetic analysis. Lancet Oncol. 2012; 13:607–15. 10.1016/S1470-2045(12)70137-7 22575588

[25] Hong ST, Fang Y. *Clonorchis sinensis* and clonorchiasis, an update. Parasitol Int. 2012; 61:17–24. 10.1016/j.parint.2011.06.007 21741496

[26] Tantrawatpan C, Intapan PM, Thanchomnang T, Sanpool O, Janwan P, Lulitanond V, et al Development of a PCR assay and pyrosequencing for identification of important human fish-borne trematodes and its potential use for detection in fecal specimens. Parasit Vectors. 2014; 7:88 10.1186/1756-3305-7-88 24589167 PMC3943809

[27] Chai JY, Lee SH. Intestinal trematodes of humans in Korea: Metagonimus, heterophyids and echinostomes. Korean J Parasitol. 1990; 28: 103–22. 10.3347/kjp.1990.28.suppl.103 2133416

[28] Chai JY, Song TE, Han ET, Guk SM, Park YK, Choi MH, et al Two endemic foci of heterophyids and other intestinal fluke infections in southern and western coastal areas in Korea. Korean J Parasitol. 1998; 36:155–61. 10.3347/kjp.1998.36.3.155 9755586 PMC2732926

[29] Lee SH, Chai JY, Hong ST. *Gymnophalloides seoi* n. sp. (Digenea: Gymnophallidae), the first report of human infection by a gymnophallid. J Parasitol. 1993; 79:677–80. 8410538

[30] Sarafraz S, Farajnia S, Jamali J, Khodabakhsh F, Khanipour F. Detection of *Dientamoeba fragilis* among diarrheal patients referred to Tabriz health care centers by nested PCR. Trop Biomed. 2013; 30:113–8. 23665716

