# Supplementary figures and images for: Multiplex Real-Time PCR Assay Targeting Eight Parasites Customized to the Korean Population: Potential Use for Detection in Diarrheal Stool Samples from Gastroenteritis Patients
Source: PLoS One. 2016 Nov 18;11(11):e0166957. doi: 10.1371/journal.pone.0166957 (PMC5115832; doi:10.1371/journal.pone.0166957)

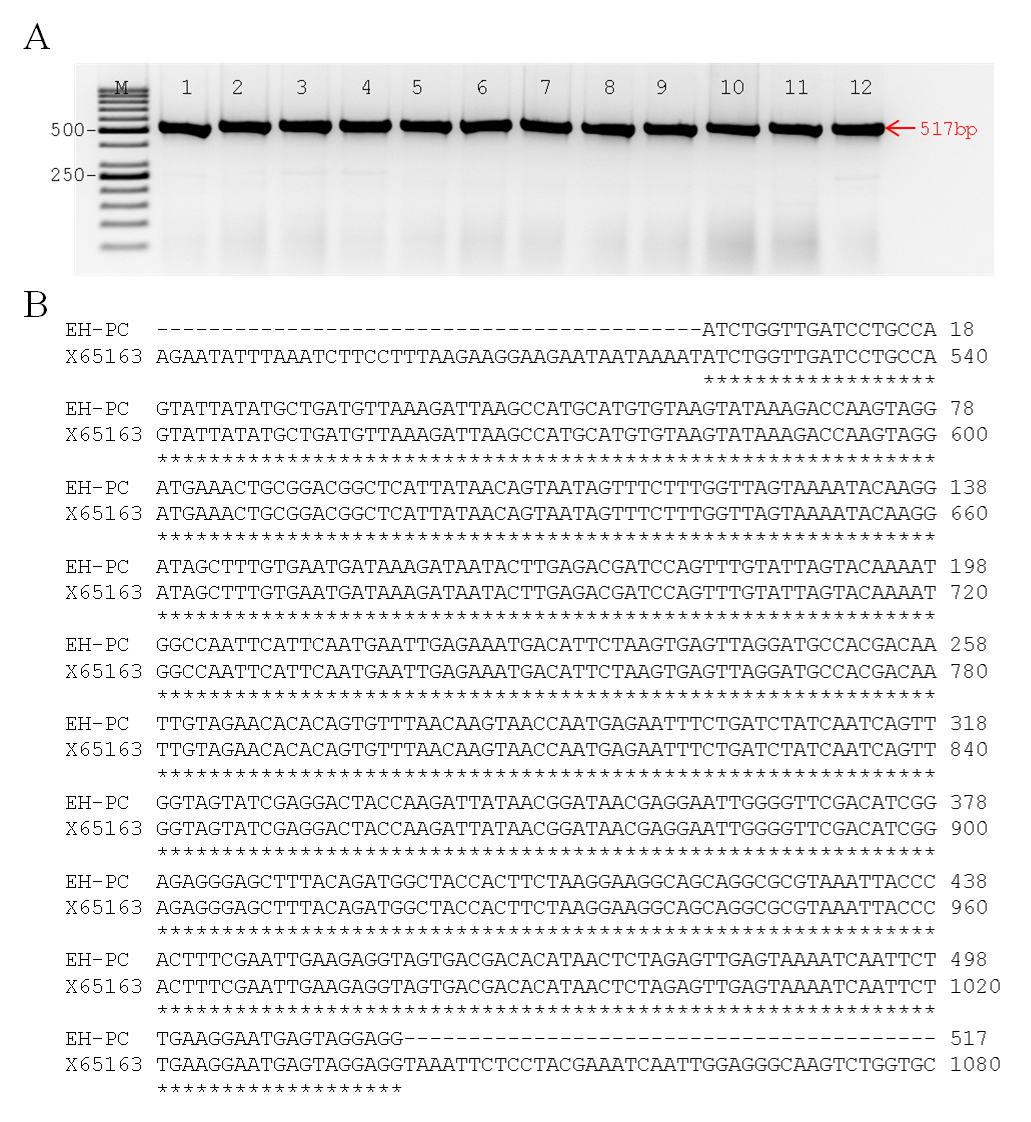

Supplement: S1 Fig — PCR products (517 bp size) obtained from 12 cloned colonies (A) and their sequencing results showing 100% match with sequences of E. histolytica (GenBank: X65163.1). (TIF) [file pone.0166957.s001.tif]

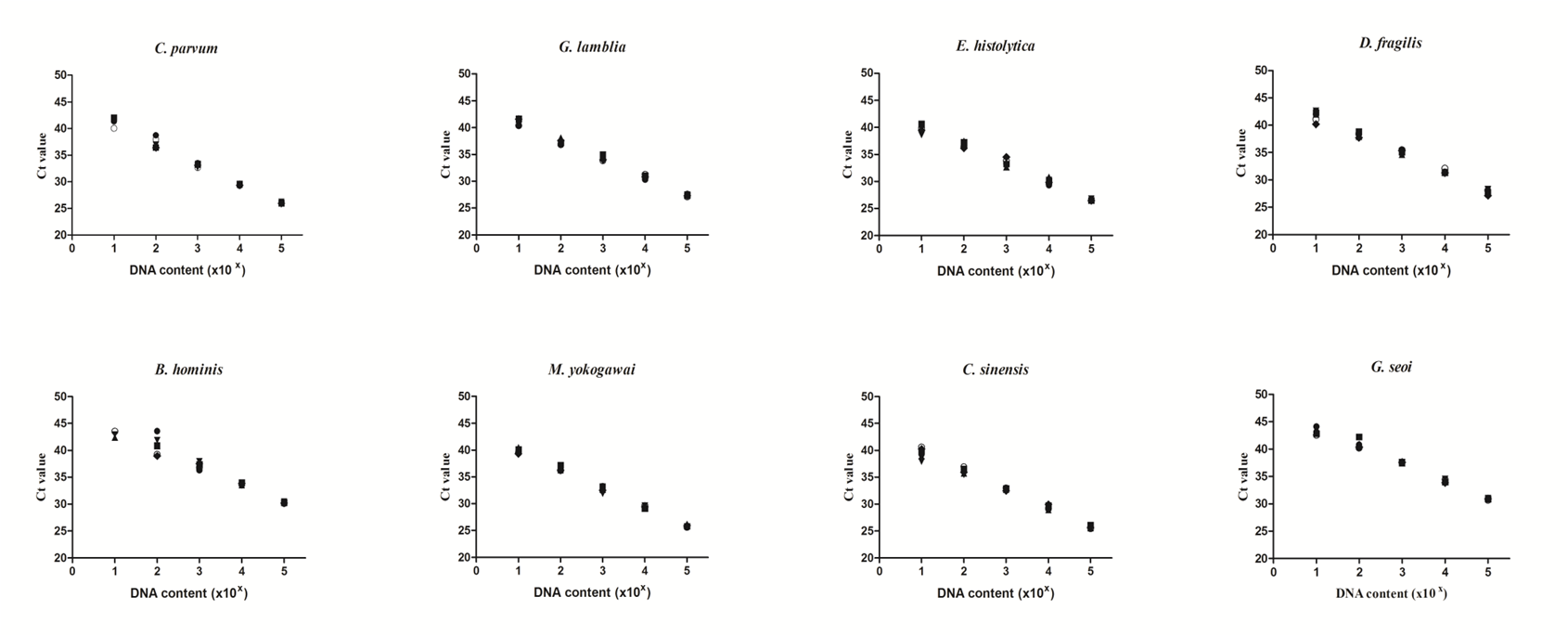

Supplement: S2 Fig — PCRs were performed on six 10-fold dilutions from 100 fg to 10 ag. Good correlations between the DNA concentration and the threshold cycle (Ct) values are seen. (TIF) [file pone.0166957.s002.tif]
