# Supplementary material for: Multiplex Real-Time PCR Assay Targeting Eight Parasites Customized to the Korean Population: Potential Use for Detection in Diarrheal Stool Samples from Gastroenteritis Patients
Source: PLoS One. 2016 Nov 18;11(11):e0166957. doi: 10.1371/journal.pone.0166957 (PMC5115832; doi:10.1371/journal.pone.0166957)
Supplement: S1 Table — (PDF) [file pone.0166957.s003.pdf]

S1 Table. Details of amplification primers of the parasites for making positive controls

Target organism	Forward primer sequence (5'-3')	Reverse primer sequence (5'-3')	BLAST identity (%)	Target	Accession number of target sequence
Gymnophalloides seoi	TTTTGCCTGGTTTGGAATAGTT	GCCACCACAAATCAAGTATCA	380/380 (100%)	CO1	AF096234.3
Blastocystis hominis	GCTCGTCTCAAAGATTAAG	TGCCCTCCAATTGTTTATCG	511/511 (100%)	18s rRNA	EU482085.1
Cryptosporidium parvum	TGTTGCATTCACTATGCCTGA	GGACTGAAATACAGGCATTATCTTG	533/533 (100%)	Cowp 1	AB089292.1
Giardia lamblia	AGATGATCA AGGACGCCATC	GACGAGCTTCGTGTTTGTGA	464/464 (100%)	beta-giardin	XM_001705373.1
Entamoeba histolytica	ATCTGGTTGATCCTGCCAGT	CCTCCTACTCATTCCTTCAAGA	540/540 (100%)	18s rRNA	X65163.1
Metagonimus yokogawai	GTTGATTCTCCCAGGGGTTT	CCATTATGGAGGCCGATAAG	360/360 (100%)	CO1	AB470519.1
Clonorchis sinensis	GGTGGTTTGAGCTCATCATATGT	CGAGTTCCAGCAAGCATATATAATC	360/360 (100%)	CO1	FJ381664.2

CO1 = cytochrome c oxidase subunit 1; Cowp = Cryptosporidium oocyst wall protein; DNA synthesis for *Dientamoeba fragilis* (GenBank: JQ677163.1) was requested to the Bioneer (Bioneer, Korea).
