# Supplementary material for: Multiplex Real-Time PCR Assay Targeting Eight Parasites Customized to the Korean Population: Potential Use for Detection in Diarrheal Stool Samples from Gastroenteritis Patients
Source: PLoS One. 2016 Nov 18;11(11):e0166957. doi: 10.1371/journal.pone.0166957 (PMC5115832; doi:10.1371/journal.pone.0166957)
Supplement: S2 Table — (PDF) [file pone.0166957.s004.pdf]

S2 Table. The cross-reactivity test of the multiplex real-time PCR assay in relation to various microorganisms including 27 bacteria/candida, 11 parasites, and 16 viruses

**No.**	**Organisms**	**Name**	**Results**	**No.**	**Organisms**	**Name**	**Results**
**1**	Bacteria	*Clostridium difficile*	U.D.	28	Parasites	*Ascaris lumbricoides*	U.D.
**2**		*Yersinia enterocolitica*	U.D.	29		*Enterobius vermicularis*	U.D.
**3**		*Salmonella typhimurium*	U.D.	30		*Taenia asiatica*	U.D.
**4**		*Proteus mirabilis*	U.D.	31		*Taenia solium*	U.D.
**5**		*Pseudomonas aeruginosa*	U.D.	32		*Taenia saginata*	U.D.
**6**		*Escherichia coli*	U.D.	33		*Diphyllobothrium nihonkaiense*	U.D.
**7**		*Lactobacillus acidophilus*	U.D.	34		*Plasmodium falciparum*	U.D.
**8**		*Bifidobacterium bifidum*	U.D.	35		*P. vivax*	U.D.
**9**		*Enterobacter aerogenes*	U.D.	36		*P. ovale*	U.D.
**10**		*Clostridium perfringens*	U.D.	37		*P. malariae*	U.D.
**11**		*Enterococcus faecalis*	U.D.	38		*T. vaginalis*	U.D.
**12**		*Shigella flexneri*	U.D.	39	Viruses	Noro virus	U.D.
**13**		*Klebsiella pneumoniae*	U.D.	40		Rota virus	U.D.
**14**		*Campylobacter jejuni*	U.D.	41		HSV1	U.D.
**15**		*Candida albicans*	U.D.	42		HSV-2	U.D.
**16**		*Filobasidiella neoformans*	U.D.	43		HAV	U.D.
**17**		*Staphylococcus epidermidis*	U.D.	44		HBV	U.D.
**18**		*Aspergillus fumigatus*	U.D.	45		HCV	U.D.
**19**		*Staphylococcus aureus*	U.D.	46		EBV	U.D.
**20**		*Mycobacterium tuberculosis*	U.D.	47		JEV	U.D.
**21**		*M. intracellulare*	U.D.	48		Enterovirus 70	U.D.
**22**		*M. abscessus*	U.D.	49		Enterovirus 71	U.D.
**23**		*M. hominis*	U.D.	50		BKV	U.D.
**24**		*Chlamydia trachomatis*	U.D.	51		Dengue virus 2	U.D.
**25**		*Neisseria gonorrhoeae*	U.D.	52		Dengue virus 3	U.D.

**26**		*Ureaplasma parvum*	U.D.	53		Dengue virus 4	U.D.
**27**		*U. urealyticum*	U.D.	54		Human adenovirus	U.D.

U.D. = undetected
