# Supplementary material for: Multiplex Real-Time PCR Assay Targeting Eight Parasites Customized to the Korean Population: Potential Use for Detection in Diarrheal Stool Samples from Gastroenteritis Patients
Source: PLoS One. 2016 Nov 18;11(11):e0166957. doi: 10.1371/journal.pone.0166957 (PMC5115832; doi:10.1371/journal.pone.0166957)
Supplement: S3 Table — (PDF) [file pone.0166957.s005.pdf]

S3 Table. **Interference analysis involving addition of different concentrations of interferents to positive controls**

Interferents	Cryptosporidium parvum	Giardia lamblia	Entamoeba histolytica	Dientamoeba fragilis	Blastocystis hominis	Clonorchis sinensis	Metagonimus yokogawai	Gymnophalloides seoi
	C_t values of parasite PCR after addition of various concentrations of interferents							
**Erythrocytes**								
**20%**	U.D.	U.D.	U.D.	U.D.	U.D.	U.D.	U.D.	U.D.
**4%**	34.06	36.92	36.55	34.65	36.44	32.27	35.15	37.45
**1%**	34.18	36.21	36.57	35.32	36.76	32.32	35.43	37.31
**Negative**	33.87	36.01	36.11	35.15	36.51	32.2	35.76	37.04
**Leucocytes**								
**20%**	U.D.	U.D.	U.D.	U.D.	U.D.	U.D.	U.D.	U.D.
**4%**	33.3	34.21	33.32	34.9	37.58	31.76	32.04	37.31
**1%**	32.63	33.84	33.1	34.93	37.69	31.8	32.12	37.47
**Negative**	33.09	34.06	33.24	34.7	37.65	31.96	32.05	37.26
**Bilirubin**								
**2 μg/mL**	32.94	34.56	36.6	34.84	37.23	36.49	33.82	37.48
**1 μg/mL**	32.72	37.45	36.44	34.06	37.22	36.44	34.06	36.64
**0.5 μg/mL**	32.75	37.31	36.76	34.18	37.09	36.76	34.18	37.46
**Negative**	32.91	37.04	36.51	34.12	37.18	36.51	33.87	37.19
**Bile salts**								
**1 mg/mL**	32.51	34.76	33.28	34.89	37.31	37.48	33	37.49
**0.5 mg/mL**	32.81	34.44	32.78	34.96	37.45	36.64	32.49	37.28
**0.1 mg/mL**	33.03	34.19	33.37	34.81	37.38	37.46	32.37	37.54
**Negative**	32.49	34.27	32.66	35.13	37.61	37.19	32.71	37.37
**Heparin**								

	**0.2 IU**	38.05	U.D.	U.D.	U.D.	U.D.	U.D.	37.51	39.86
	**0.1 IU**	32.73	33.47	33.35	33.3	37.13	32.56	32.2	36.75
	**0.05 IU**	32.8	33.43	33.32	33.16	36.51	32.34	31.71	37.33
	**Negative**	32.62	33.35	32.62	33.04	36.49	32.11	31.31	37.31
**Acetaminophen**									
	**200 μg/mL**	34.12	34.27	32.98	34.75	33.71	32.62	32.73	33.66
	**100 μg/mL**	32.64	33.53	33.14	34.79	32.84	32.47	31.99	33.74
	**50 μg/mL**	32.42	32.88	33.2	33.37	32.46	30.02	31.29	33.37
	**Negative**	32.71	32.52	33.73	32.95	32.38	32.81	32.85	32.98
**Ibuprofen**									
	**200 μg/mL**	34.12	34.27	32.98	34.75	33.71	32.62	32.73	33.66
	**100 μg/mL**	32.64	33.53	33.14	34.79	32.84	32.47	31.99	33.74
	**50 μg/mL**	32.42	32.88	33.2	33.37	32.46	30.02	31.29	33.37
	**Negative**	32.71	32.52	33.73	32.95	32.38	32.81	32.85	32.98

U.D. = undetected
