# Supplementary material for: Multiplex Real-Time PCR Assay Targeting Eight Parasites Customized to the Korean Population: Potential Use for Detection in Diarrheal Stool Samples from Gastroenteritis Patients
Source: PLoS One. 2016 Nov 18;11(11):e0166957. doi: 10.1371/journal.pone.0166957 (PMC5115832; doi:10.1371/journal.pone.0166957)
Supplement: S4 Table — (PDF) [file pone.0166957.s006.pdf]

**S4 Table. Sensitivity and specificity of microscopic examination and multiplex qPCR in detection of the targeted parasites in this study**

| **Final identification** | **No. of positive specimens** | **Microscopy** | | **Multiple qPCR** | |
|---|---|---|---|---|---|
| | | **Sensitivity (%) [95% confidence interval]** | **Specificity (%) [95% confidence interval]** | **Sensitivity (%) [95% confidence interval]** | **Specificity (%) [95% confidence interval]** |
| *Blastocystis hominis* | 8 | 0% [0.0-36.94] | 100% [96.84-100.0] | 100% [63.06-100.0] | [96.84-100.0] |
| *Cryptosporidium parvum* | 1 | 0% [0.0-97.50] | 100% [97.02-100.0] | 100% [2.50-100.0] | [97.02-100.0] |
| *Clonorchis sinensis* | 2 | 50% [1.26-98.74] | 100% [97.00-100.0] | 100% [15.81-100.0] | [97.00-100.0] |
| *Entamoeba histolytica* [a] | 2 | 100% [15.81-100.0] | 100% [97.00-100.0] | 100% [15.81-100.0] | [97.00-100.0] |
| *Giardia lamblia* [a] | 2 | 100% [15.81-100.0] | 100% [97.00-100.0] | 100% [15.81-100.0] | [97.00-100.0] |
| *Gymnophalloides seoi* [a] | 2 | 0% [0.0-84.19] | 100% [97.00-100.0] | 100% [15.81-100.0] | [97.00-100.0] |
| *Metagonimus yokogawai* | 0 | N.A. | 100% [97.00-100.0] | N.A. | [97.00-100.0] |
| *Dientamoeba fragilis* | 0 | N.A. | 100% [97.00-100.0] | N.A. | [97.00-100.0] |

[a] The six positive controls include 2 of *Entamoeba histolytica*, 2 of *Gilardia lamblia*, and 2 of *Gymnophalloides seoi*; N.A. = not available.
